# The Practitioners' Bookshelf

**Published:** 1892-05-21

**Authors:** 


### PRACTITIONERS' BOOKSHELF.

### A MEDICAL YEAR BOOK.*

The issue for this year is as replete with interest and useful information as the perusal of its predecessors would lead one to predict. The general scheme of the book is on the same lines as heretofore, though one or two new additions have been made to the staff of contributors, which is now largely composed of members of St. Mary's Hospital. Though this may have its advantages in the matter of uniformity of style and continuity in method, it lays itself open to the objection of narrowness and restriction in scope, and, moreover, loses the advantages which the experience of other medical centres might suggest in the compilation of a work which is supposed to be entirely representative of all sides of medical science.

With regard to the various sections in this work, that on Tuberculosis is a valuable summary of the successes and failures of Koch's method of treatment, the controversy on which subject will ever make the year 1891 memorable in the annals of medical literature. A careful perusal of the forty pages which are devoted to this subject will leave the reader in no doubt that this form of treatment in its present state of development is a total and entire failure.

The section on Gout contains an account of the properties and uses of a new drug, which goes by the name of piperazine. It has the valuable property of forming an exceedingly soluble salt when combined with uric acid, being nearly ten times as soluble as the most soluble of the known metallic urates, namely, that of lithium. It is usually prescribed in doses of thirty grains three times a day, and in most cases the result is highly satisfactory. The effect in cases of gravel is very marked, this symptom being either materially relieved or else entirely removed. In this article, as well as in several others, it is a source of annoyance to the reader, as well as the practitioner, to find that the doses are given in grams, instead of the more familiar grains of the British Pharmacopæia. A very slight extra trouble on the part of the translators of these articles would obviate this source of annoyance.

To the subject of Diabetes, the pathology of which becomes more obscure the further it is investigated, is allotted several pages by Dr. Ralfe, who treats of this section. In it we learn that Dr. Schmitz believes that a pre-disposition must pre-exist, and, like phthisis, he holds it to be infectious, and possibly due to a germ, inasmuch as he believes that it can be communicated from husband to wife, and because it is much more prevalent in certain districts that in others. In Malta, for instance, it is more fatal than tuberculosis is in Germany. Dr. Wright's paper, which appeared in the *British Medical Journal* in the early part of last year, is given *in extenso* in this article. The use of lævo rotatory sugars, such as those found in Jerusalem artichokes and in dahlia tubers, is highly recommended, since this form of sugar appears to undergo oxidation in the tissues, and does not appear as such in the urine of the diabetic. The pancreas, as in the "Year Book" for 1891, is made responsible for a large number of cases of diabetes, and, possibly, in the next addition, the disease will be classed amongst those of the pancreas.

* "The Year Book of Treatment for 1892." 3/6. (Cassell and Company.)

---

The decrease of population in France, which is exercising the minds of many in that country, has had some light thrown upon its causes by M. L. Landouzy. His investigations into the effect of tuberculosis upon the mortality of infants, have led him to the conclusion that this disease depopulates the country to a far greater extent than alcoholism or Malthusianism combined. According to the Professor's showing, nothing is done in France to place people on their guard against the contagion of tuberculosis. His work, which has attracted much attention in Paris, is to be published in English.

